# Ovariectomy-Induced Hepatic Lipid and Cytochrome P450 Dysmetabolism Precedes Serum Dyslipidemia

**DOI:** 10.3390/ijms22094527

**Published:** 2021-04-26

**Authors:** Hana Malinská, Martina Hüttl, Denisa Miklánková, Jaroslava Trnovská, Iveta Zapletalová, Martin Poruba, Irena Marková

**Affiliations:** 1 Centre for Experimental Medicine, Institute for Clinical and Experimental Medicine, 14021 Prague, Czech Republic; martina.huttl@ikem.cz (M.H.); denisa.miklankova@ikem.cz (D.M.); jaroslava.trnovska@ikem.cz (J.T.); irma@ikem.cz (I.M.); 2 Department of Pharmacology, Faculty of Medicine and Dentistry, Palacky University, 77900 Olomouc, Czech Republic; iveta.zapletalova@upol.cz (I.Z.); martin.poruba@upol.cz (M.P.)

**Keywords:** ovariectomy, metabolic syndrome, brown adipose tissue, hepatic steatosis, obesity, insulin resistance, methylglyoxal

## Abstract

Ovarian hormone deficiency leads to increased body weight, visceral adiposity, fatty liver and disorders associated with menopausal metabolic syndrome. To better understand the underlying mechanisms of these disorders in their early phases of development, we investigated the effect of ovariectomy on lipid and glucose metabolism. Compared to sham-operated controls, ovariectomized Wistar female rats markedly increased whole body and visceral adipose tissue weight (*p* ˂ 0.05) and exhibited insulin resistance in peripheral tissues. Severe hepatic triglyceride accumulation (*p* ˂ 0.001) after ovariectomy preceded changes in both serum lipids and glucose intolerance, reflecting alterations in some CYP proteins. Increased CYP2E1 (*p* ˂ 0.05) and decreased CYP4A (*p* ˂ 0.001) after ovariectomy reduced fatty acid oxidation and induced hepatic steatosis. Decreased triglyceride metabolism and secretion from the liver contributed to hepatic triglyceride accumulation in response to ovariectomy. In addition, interscapular brown adipose tissue of ovariectomized rats exhibited decreased fatty acid oxidation (*p* ˂ 0.01), lipogenesis (*p* ˂ 0.05) and lipolysis (*p* ˂ 0.05) despite an increase in tissue weight. The results provide evidence that impaired hepatic triglycerides and dysregulation of some CYP450 proteins may have been involved in the development of hepatic steatosis. The low metabolic activity of brown adipose tissue may have contributed to visceral adiposity as well as triglyceride accumulation during the postmenopausal period.

## 1. Introduction

Postmenopausal women are highly susceptible to metabolic syndrome, type 2 diabetes and cardiovascular disease [1,2]. Prevalence of metabolic syndrome in postmenopausal women is 60% compared to 20% in premenopausal women. The postmenopausal period is frequently accompanied by increased visceral adiposity, insulin resistance, fatty liver and disorders of the lipid and glucose metabolism. Higher prevalence of these disorders after menopause suggests that disruption of ovarian function may contribute to the incidence of these conditions. However, the underlying mechanisms remain unclear.

Estrogens play key roles in protecting against obesity and are important regulators of several metabolic processes, including glucose and lipid metabolism, body weight, adipose tissue distribution, caloric intake and energy expenditure [3].

Ovarian hormones contribute to the maintenance of insulin sensitivity in white adipose tissue (WAT), preventing low-grade inflammation of WAT [4] independently of energy status. Although estradiol deficiency slightly increases food intake, it does not seem to be the primary cause of obesity in ovariectomized (OVX) female rats [5]. Alterations in glucose and lipid homeostasis may precede increased body weight gain after menopause.

Increasing evidence indicates that brown adipose tissue (BAT) has a critical role in controlling energy metabolism, an activity possibly regulated by sexual hormones [6]. The thermogenic ability of BAT has prompted research into the positive influences of BAT on body weight regulation and glucose and lipid homeostasis [7]. One proposed therapeutic strategy involves augmenting the oxidative metabolism of BAT [8].

Postmenopausal women display increased incidence of hepatic steatosis and disrupted lipid homeostasis [9]. Dysregulated lipid metabolism in turn affects body fat mass, adiposity, fatty acid metabolism and the basal metabolic rate. Via effects on hepatic lipid metabolism, regulation and transport, estrogen signaling in the liver is important in preventing the development of hepatic steatosis. Steroid hormones including estrogens and androgens are understood to regulate the expression and activity of enzymes involved in lipid metabolism in the liver. The cytochrome P450 (CYP) protein family contains important enzymes involved in xenobiotics, endogenous compound metabolism and lipid homeostasis. While CYP2E1 contributes to fatty acid oxidation, CYP4A catalyzes omega hydroxylation of the endogenous fatty acids and prostaglandins that regulate many physiological functions [10,11]. Changes in the gene regulation and expression of CYPs likely reflect the early stage of NAFLD and may be beneficial in treating hepatic steatosis.

Some of the factors involved in the pathogenesis of hepatic steatosis include hepatic insulin resistance, the pro-inflammatory pathway and oxidative as well as (recently) carbonyl stress linked to increased production of dicarbonyls in the liver. Increased accumulation of dicarbonyls like methylglyoxal may increase oxidative stress in the liver, activate the inflammatory pathway and play a key role in the development of other metabolic disorders associated with postmenopausal metabolic syndrome [12].

However, the exact mechanisms involved in the early stages of ovariectomy-induced lipid dysmetabolism, including their relations to circulating serum lipids and whole body energy metabolism, have yet to be established. To better understand the underlying mechanisms of disorders associated with ovariectomy-induced metabolic syndrome, we examined the effect of ovariectomy on tissue metabolic disturbances linked to metabolic syndrome. We hypothesized that ovariectomy leads to hepatic lipid accumulation and impaired cytochrome P450 family proteins.

## 2. Results

Before ovariectomy and upon starting the trial, no differences in initial body weight (243 ± 18 vs. 253 ± 7 g), glucose (5.0 ± 0.1 vs. 5.2 ± 0.2 mmol/L) or serum triglycerides (2.00 ± 0.36 vs. 1.81 ± 0.27 mmol/L) were observed between the experimental groups.

### 2.1. Effects of Ovariectomy on Body Weight, Adiposity, Glucose Tolerance and Insulin Sensitivity

**Figure 1:**
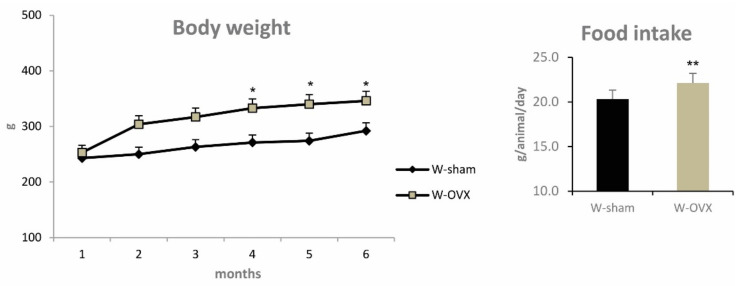
Whole body weight (over a 4-month experimental period) and food intake in ovariectomized Wistar female rats compared to sham-operated controls. Significance was determined using the unpaired Student’s *t* test (* denotes *p* < 0.05, ** denotes *p* < 0.01).

As expected, ovariectomy in female Wistar rats was associated with decreased serum levels of the sex hormones 17β-estradiol and 17β-hydroxyprogesterone (Table 1). In keeping with the inhibitory effect of estradiol on appetite, ovariectomized female rats exhibited higher food intake (+9%, *p* ˂ 0.01) (Figure 1) and markedly increased serum levels of leptin. No changes in the appetite hormone ghrelin were observed (Table 1).

**Figure 2:**
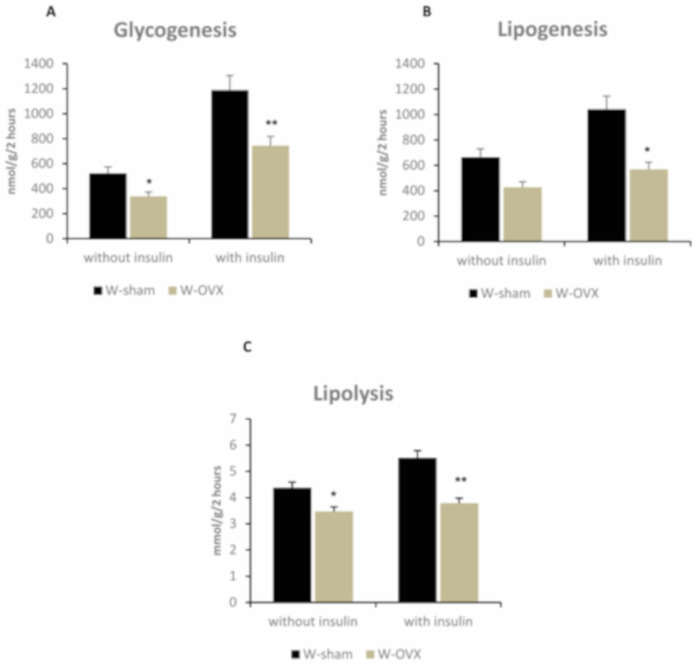
Basal and insulin-stimulated sensitivity in skeletal muscle (**A**-glycogenesis) and basal and insulin-stimulated sensitivity in perimetrial adipose tissue (**B**-lipogenesis). Basal and adrenaline-stimulated lipolysis from perimetrial adipose tissue (**C**) in ovariectomized Wistar female rats compared to sham-operated controls. Significance was determined using the unpaired Student’s *t* test (* denotes *p* < 0.05, ** denotes *p* < 0.01).

Compared with sham-operated controls, ovariectomized female rats exhibited markedly increased body weight (+10%, *p* ˂ 0.05) and visceral adipose tissue weight (+10%, *p* ˂ 0.05) (Table 1 and Figure 1). Insulin sensitivity of perimetrial adipose tissue measured according to the incorporation of glucose into lipids was significantly decreased in ovariectomized female rats compared to sham controls (*p* ˂ 0.05). Basal and insulin-stimulated incorporation of glucose into glycogen measured in the soleus muscle as a parameter of muscle tissue insulin sensitivity was significantly reduced in ovariectomized female rats compared to controls (*p* ˂ 0.01) (Figure 2). Despite increased body weight and impaired insulin sensitivity in skeletal muscle and visceral adipose tissue, ovariectomized female rats exhibited no changes in fasting serum glucose, AUC_0-120_, insulin, HMW adiponectin or pro-inflammatory hs-CRP in comparison with W-sham rats (Table 1). On the other hand, serum levels of pro-inflammatory chemokine MCP-1 was elevated in OVX females. Ovariectomized rats showed elevated serum NEFA levels and triglyceride accumulation in skeletal muscle, which could contribute to insulin resistance in this tissue (Table 2). As shown in Figure 2, ovariectomized female rats displayed decreased basal as well as adrenaline-stimulated lipolysis. The observed increase in serum levels of pro-inflammatory MCP-1 and the decrease in proteins in perimetrial adipose tissue (0.800 ± 0.075 vs. 1.136 ± 0.171%, *p* ˂ 0.001) may point to the early development of adipose tissue insulin resistance.

### 2.2. Effects of Ovariectomy on BAT Metabolic Activity

**Figure 3:**
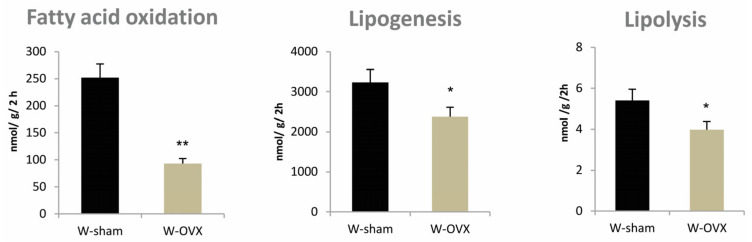
Substrate utilization in interscapular brown adipose tissue: fatty acid oxidation, lipogenesis and lipolysis in ovariectomized Wistar female rats compared to sham-operated controls. Significance was determined using the unpaired Student’s *t* test (* denotes *p* < 0.05, ** denotes *p* < 0.01).

As shown in Figure 3, interscapular BAT of ovariectomized female rats exhibited markedly reduced fatty acid oxidation (−72%, *p* < 0.01), lipogenesis (−26%, *p* < 0.05) and lipolysis (−25%, *p* < 0.05) compared to sham controls. Metabolic activity of interscapular BAT was markedly reduced despite significantly increased interscapular BAT (*p* < 0.05) in ovariectomized female rats.

### 2.3. Effects of Ovariectomy on Serum and Hepatic Lipid Regulation and Transport

**Figure 4:**
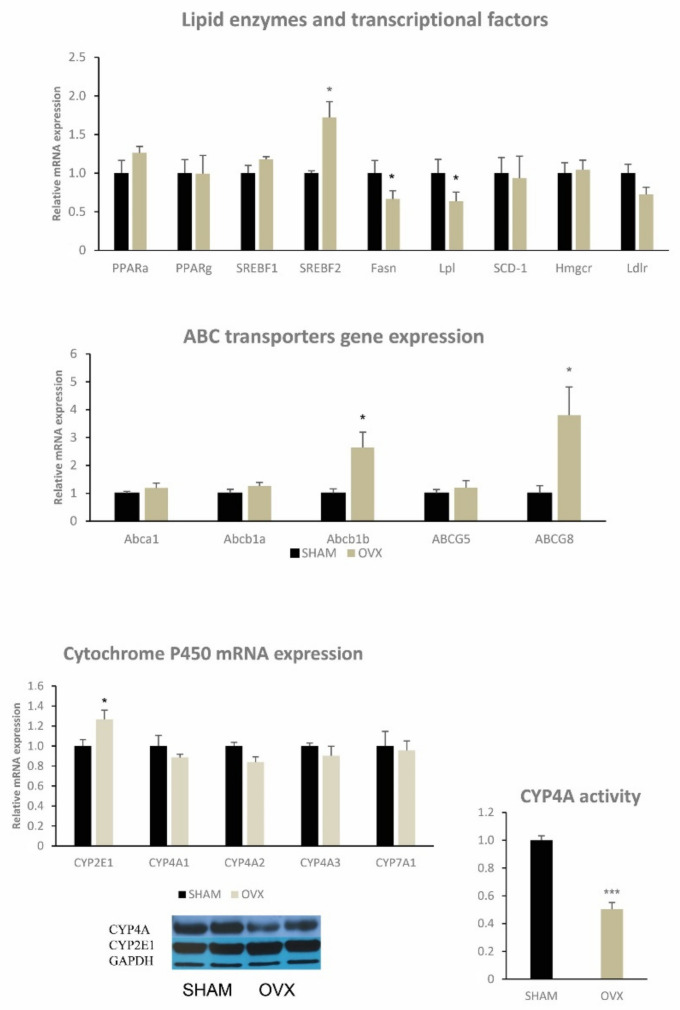
Relative mRNA expression of enzymes and transcriptional factors involved in lipid metabolism and ABC transporters in the liver. Relative mRNA expression, proteins and activity of some CYP450 family proteins in the liver in ovariectomized Wistar female rats compared to sham-operated controls. Significance was determined using the unpaired Student’s *t* test (* denotes *p* < 0.05, *** denotes *p* < 0.001); PPAR—peroxisome proliferator-activated receptor, SREBF—sterol regulatory element-binding protein, FAS—fatty acid synthase, LPL—lipoprotein lipase, SCD1—stearoyl CoA desaturase-1, HMGCR—hydroxyl-methyl-glutaryl-CoA reductase, LDLR—low-density lipoprotein receptor.

Although no changes in hepatic cholesterol levels were observed, ovariectomy in female rats markedly increased ectopic triglyceride accumulation in the liver (*p* < 0.001) (Table 2), perhaps highlighting its involvement in the menopausal development of hepatic steatosis and other dysfunctions in the liver. However, there were no differences in serum triglycerides and cholesterol levels between OVX and sham-controlled female rats (Table 1). On the other hand, ovariectomized female rats exhibited increased levels of HDL cholesterol, which correlated with increased expression of *Abca1* (Table 1 and Figure 4).

As shown in Figure 4, ovariectomized female rats exhibited significant changes in two cytochrome P450 enzymes—CYP2E1 and CYP4A—in the liver. Although hepatic gene expression of *Cyp4a* was not influenced by ovariectomy, the protein content and activity of this enzyme was significantly decreased. Hepatic mRNA and protein content of CYP2E1 increased by 27% and 10%, respectively. Gene expression of the hepatic transporter *Abcg8* and transcriptional factor *Srebf2* were significantly increased, which shows that ovariectomy affects the metabolism and elimination of cholesterol (Figure 4). On the other hand, hepatic gene expression of *Fas* and *Lpl*-reducing fatty acids as well as triglyceride metabolism/turnover were decreased (Figure 4). Genes of other enzymes and receptors involved in cholesterol and fatty acid metabolism such as *Pparα Pparγ*, *Srebf1*, *Scd1*, *Hmgcr*, *Abcb1a*, *Abcb1b*, *Abcg5* and *Ldlr* were not significantly affected.

### 2.4. Effects of Ovariectomy on Oxidative and Dicarbonyl Stress in the Liver

**Figure 5:**
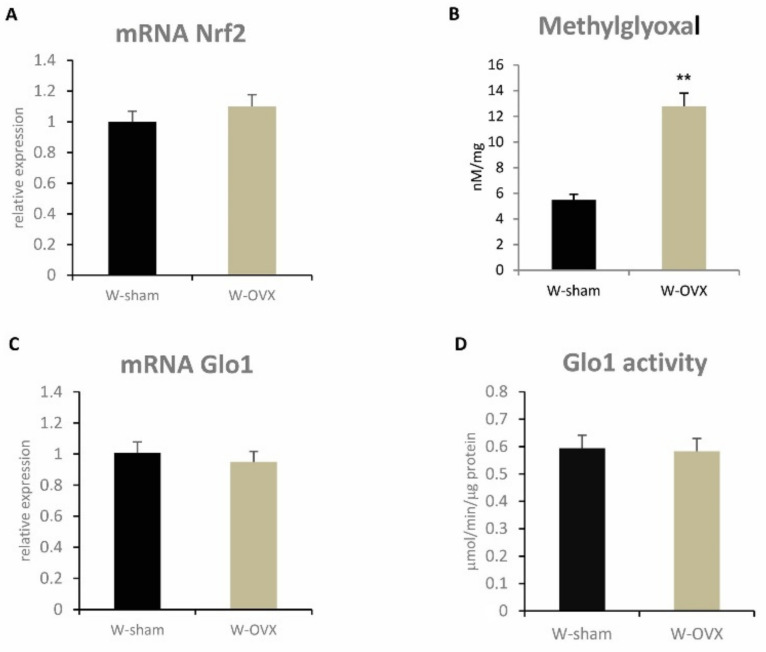
Hepatic parameters of oxidative and dicarbonyl stress: relative mRNA expression of NRF2 (**A**), methylglyoxal concentration (**B**), mRNA expression and activity of Glo1 (**C**,**D**) in ovariectomized Wistar female rats compared to sham-operated controls. Nrf2—nuclear factor-erythroid 2-related factor-2, Glo1—glyoxalase-1. Significance was determined using the unpaired Student’s *t* test (** denotes *p* ˂ 0.01).

The marked accumulation of hepatic triglycerides in ovariectomized female rats was associated with significantly elevated hepatic levels of methylglyoxal (*p* ˂ 0.01) (Figure 5), a reactive dicarbonyl compound that contributes to the development of liver steatosis in postmenopausal metabolic syndrome. However, there were no differences in either gene expression or activity of glyoxalase-1 (Glo1), an enzyme that participates in methylglyoxal detoxification. In addition, ovariectomy led to impaired balance in reduced and oxidized forms of glutathione in the liver (GSH/GSSG *p* ˂ 0.01) (Table 2), which may have increased oxidative as well as dicarbonyl stress. Reduced hepatic levels of glutathione were associated with significantly decreased activity of the glutathione-dependent antioxidant enzyme GPx (Table 2). Gene expression of *Nrf2/Nfe2l2* (nuclear factor-erythroid 2-related factor 2), a key transcription factor responsible for constitutive and inducible expression of ARE-regulated genes in antioxidant enzymes (Figure 5), was unchanged after ovariectomy.

Severe triglyceride accumulation and changes in oxidative and dicarbonyl stress in the liver after ovariectomy were associated with elevated hepatic enzyme activity of AST and GGT (Table 2).

## 3. Discussion

Although prevalence of metabolic syndrome is generally lower in women than in men [13], it is a greater risk factor for women in cases where MS has yet to manifest. The risk of women developing MS [1] and cardiovascular disease [2] increases dramatically with both age and loss of ovarian function.

The role of sexual hormones in modulating lipid metabolism, glucose tolerance and insulin sensitivity is of great interest due to the higher risk of metabolic syndrome development after menopause. Early events in metabolic dysregulation associated with ovarian hormone deficiency are understood to be important for establishing dietary and pharmacological targets after menopause.

### 3.1. Body Weight

In the present study, ovariectomy-induced whole-body and visceral adipose tissue weight gain was associated with slightly increased food intake (9%). This effect tallies with previous rodent studies [14] and human studies of postmenopausal women [15] that have examined the role of ovarian hormones in controlling energy metabolism. An increase in food intake can also occur due to raised levels of appetite hormones responsible for regulating feelings of satiety such as leptin. Circulating levels of leptin after ovariectomy were observed in this study. However, another study of ovariectomized female rats [5] showed that although food intake slightly increased after ovariectomy, the increase applied only to fecal quantities, with no differences in the feces-to-food intake ratio. These findings support the hypothesis that metabolic changes after ovariectomy affect body weight gain more than a slight increase in food intake.

### 3.2. BAT

Ovarian hormones are understood to regulate body fat distribution, with white and brown adipose tissues both affected. Decreased metabolic activity of BAT can also contribute to body weight gain after ovariectomy, first manifesting in a noticeable reduction of fatty acid oxidation. Several human studies have shown that BAT negatively correlates with high BMI and fat content, supporting the argument that BAT is important in preventing obesity [8,16]. In the present study, ovarian hormone deficiency in ovariectomized female rats led to reduced metabolic activity, despite an increase in the relative weight of BAT. Our finding of increased BAT weight after ovariectomy, which contrasts with the observations of most other experimental studies [5], may indicate a temporary adaptive mechanism in the early development of menopause metabolic syndrome. In the human population, the transition to menopause and ovarian hormone loss are gradual, with onset usually occurring after middle age. OVX rats have been shown to exhibit a particularly marked reduction in fatty acid oxidation, which may explain the hepatic lipid accumulation observed in our study [17].

Our data on ovariectomized rats indicate that sex hormones exert profound effects on BAT activity, which supports the hypothesis that BAT influences the effect of several sex hormones on whole-body energy balance/homeostasis. While BAT mediates the ability of these hormones to modulate energy balance, estrogens as well as progesterone strongly affect BAT activity [18]. The modified interaction between BAT and sex hormones after menopause may induce obesity and other metabolic disorders associated with postmenopausal metabolic syndrome [19] such as insulin resistance and glucose intolerance [20].

### 3.3. Insulin Sensitivity and Glucose Tolerance

In the present study, although glucose tolerance was not affected after ovariectomy, decreased lipogenesis and lipolysis in BAT may have contributed to the development of insulin resistance in peripheral tissues. We observed decreased perimetrial adipose tissue and skeletal muscle insulin sensitivity in ovariectomized female rats. Not only is BAT more insulin-sensitive than WAT, but changes in the metabolic activity of BAT are understood to have systemic effects on other insulin-sensitive tissues [21]. In addition, decreased protein content in WAT may account for the replacement of small adipocytes with large, metabolically less activated adipocytes responsible for secreting pro-inflammatory adipocytokines.

The development of insulin resistance is also associated with changes to adipocytokine secretion and low-chronic inflammation in white adipose tissue. Although the relation between metabolic disorders and certain adipokines is well-established, the effect of these disorders on sex hormones is far from conclusive. In this study and other studies on rodents [22], ovarian hormone deficiency led to visceral adiposity and insulin resistance in peripheral tissues but surprisingly resulted in no changes to adiponectin levels. Human studies of postmenopausal women have produced similarly inconsistent results. While some studies have demonstrated no effect, others have reported higher levels of adiponectin in postmenopausal compared to premenopausal women adjusting for age and fat mass [23,24]. On the other hand, the markedly increased levels of leptin we observed in ovariectomized female rats suggest that the state of leptin resistance may contribute to impaired insulin sensitivity. While the results for adiponectin are contradictory, most studies of ovariectomized rodents and postmenopausal women have found markedly increased leptin levels [25,26,27].

In the present study, ectopic triglyceride accumulation in the muscles of ovariectomized rats increased, which may indicate the involvement of an important mechanism in the impairment of insulin signaling in muscles and the development of insulin resistance. Lipotoxic intermediates, primarily the diacylglycerols and ceramides generated due to accumulated lipids in muscles, interfere with and impair the insulin-signaling cascade in the PKC and PKB positions [28]. In addition, the slight elevation in circulating NEFA observed after ovariectomy in our study indicates its potential association with the development of insulin resistance, despite the reduction in lipolysis from both white and brown adipose tissue.

### 3.4. Hepatic Lipid Dysmetabolism

In this study, the marked accumulation of ectopic hepatic triglycerides after ovariectomy was not associated with changes in circulating levels of triglycerides or cholesterol. There were also no differences in cholesterol content in the liver. We therefore assume hepatic triglyceride deposition precedes changes in serum lipids as well as cholesterol dysmetabolism, representing an important early phase in the development of ovariectomy-induced hepatic steatosis. Lipid accumulation in hepatocytes is triggered by various mechanisms, including increased hepatic uptake of circulating fatty acids and de novo fatty acid synthesis along with decreased hepatic lipid oxidation and hepatic lipid export [29]. According to our results on gene expression of enzymes and transcriptional factors involved in lipid metabolism, it seems that hepatic triglyceride accumulation is probably the result of lower triglyceride turnover, reduced TG-rich VLDL secretion from the liver and lower fatty acid oxidation. We found that relative mRNA expression of *Lpl* and *Fas*, enzymes responsible for triglyceride hydrolysis and synthesis, was reduced in ovariectomized animals. Hepatic mRNA expression of *Fas*, an enzyme involved in de novo synthesis of fatty acids, was suppressed. These phenomena are probably caused by the suppressive effect of triglyceride accumulation in the liver. Similarly, gene expression of *Scd1*, a key enzyme in de novo lipogenesis, and mRNA levels of *Srebf1*, a transcriptional factor that targets important lipogenic genes, were unchanged after ovariectomy. Hepatic triglyceride accumulation due to ovariectomy occurred without affecting serum triglyceridemia, which is in agreement with previous findings on ovariectomized rodents [30].

Although some studies of ovariectomized rodents have reported increased serum cholesterol levels and liver accumulation [5,31], we found no increase in cholesterol, which suggests that hepatic triglyceride accumulation after ovariectomy precedes serum hypertriglyceridemia and hypercholesterolemia. According to our results on gene expression of enzymes and transcriptional factors involved in cholesterol metabolism, cholesterol synthesis was unchanged. However, increased gene expression of the *Abcg8* transporter indicated higher cholesterol secretion from hepatocytes into bile. ABCG5/8 transporters not only play an important role in sterol absorption and excretion but also provide an important pathway for cholesterol elimination [32,33]. While transcriptional factor SREBF1 is involved in fatty acid synthesis, SREBF2 regulates genes affecting cholesterol metabolism, the most important being HMGCR and the LDL receptor. Therefore, SREBF2 factor seems to act as an important regulatory checkpoint for controlling intracellular cholesterol homeostasis [34]. While gene expression of *Hmgcr* was unchanged, mRNA expression of LDL receptors tended to decrease after ovariectomy. These results are in agreement with a previous study that demonstrated decreased mRNA of *Ldlr* and *Pcsk9* in response to ovariectomy [35]. Higher HDL cholesterol in ovariectomized animals indicates that cholesterol efflux capacity increases and can at least partially protect against hypercholesterolemia, hepatic cholesterol accumulation and lipotoxicity [36]. Estrogens markedly influence lipid metabolism in the liver, stimulate lipid oxidation, inhibit both fatty acid uptake and triglyceride synthesis and promote lipolysis in adipose tissue [37]. In our study, estrogen deficiency after ovariectomy led to reduced lipolysis in WAT and BAT, which was surprisingly associated with slightly increased circulating NEFA. Elevated levels of NEFA can lead to decreased fatty acid oxidation in the livers of ovariectomized rats, in turn contributing to hepatic steatosis. Exacerbation of insulin resistance and hepatic lipid accumulation may underlie the predisposition for cardiovascular disease in women after menopause.

### 3.5. CYP Protein Family

Changes in the cytochrome P450 protein family (CYP2E1 and CYP4A) reflect hepatic lipid dysmetabolism and can also contribute to the early phase of ovariectomy-induced hepatic steatosis. Our results support the likelihood of hepatic steatosis development (CYP2E1) and impaired fatty acid oxidation (CYP4A).

According to recent studies, and in agreement with our findings on ovariectomized rats, the development of hepatic steatosis correlates with upregulation of CYP2E1. Reduced CYP4A activity after ovariectomy can contribute to decreased fatty acid oxidation in the liver, slightly increasing circulating NEFA as a result. The CYP4A family includes important enzymes in lipid homeostasis that catalyze omega-hydroxylation of endogenous fatty acids and prostaglandins [38]. The CYP4A enzyme family is involved in the metabolism of medium- and long-chain fatty acids, such as arachidonic and palmitic fatty acids and compounds with highly regionally selective hydroxylated terminal omega-carbon. Increased *Cyp4a* expression has been reported in ob/ob mice [39], in a diet-induced mouse model of NASH [40] and has also been detected in the livers of NAFLD patients [41]. However, CYP enzyme changes in women after menopause are currently unknown.

The effect of NAFLD/lipid accumulation in CYP450s has been studied to assess the disease-associated impact on drug metabolism and to provide recommendations for at-risk populations including postmenopausal women [42]. To date, little is known about how lipid accumulation and sex hormone deficiency after ovariectomy influence CYPs. Human CYP4A11 may serve as a mediator of oxidative stress and lipoperoxidation [11].

Our data indicate that the changes in mRNA expression of genes involved in cholesterol and lipid transport, secretion, synthesis and regulation together with changes in cytochrome P450 proteins may contribute to the altered hepatic lipid metabolism after ovariectomy. Changes to enzymes of the cytochrome P450 family should be taken into account when evaluating pharmacological interventions for women after menopause.

### 3.6. Hepatic Oxidative and Dicarbonyl Stress and Inflammation

Estradiol signaling in the liver is important for preventing diabetes and hepatic steatosis development. Estradiol is a steroid hormone that suppresses the expression of enzymes involved in lipogenesis and activates fatty acid oxidation enzymes in the liver [37].

In our study, we found that expressive hepatic triglyceride accumulation after ovariectomy was associated with markedly elevated methylglyoxal in the liver. The reduced GSH/GSSG ratio we observed may have led to oxidative and dicarbonyl stress. We assume methylglyoxal accumulation did not occur due to lower degradation but rather due to increased generation, considering Glo1 remained unchanged after ovariectomy. Dicarbonyl stress in the liver after ovariectomy is understood to contribute via several mechanisms to the development of hepatic steatosis. Methylglyoxal decreases glutathione and induces lipoperoxidation, may impair insulin signaling by inhibiting IRS1 [43], activates RAGE receptors and increases AGE production. Some inflammatory pathways, in particular those related to NFkB, are activated by methylglyoxal [44]. Elevated levels of methylglyoxal may also aggravate white adipose tissue function, which reduces adiponectin production and elevates the secretion of pro-inflammatory adipokines [45]. These results indicate that dicarbonyl stress may be involved in the pathogenesis of hepatic steatosis, oxidative stress, inflammatory pathways and hepatic insulin resistance.

## 4. Materials and Methods

### 4.1. Animals and Diet

All of the experiments were performed in agreement with the Animal Protection Law of the Czech Republic (311/1997), which is in compliance with European Community Council recommendations (86/609/ECC) for the use of laboratory animals, and were approved by the Ethics Committee of the Institute for Clinical and Experimental Medicine.

Rats were kept at a temperature of 22 °C and humidity-controlled conditions under a 12/12 h light/dark cycle with free access to a standard chow diet (23% protein, 43% starch, 7% fat, 5% fiber, 1% vitamin and mineral mixture; Bonagro, Czech Republic) and water. At the beginning of the study female rats were randomly divided into two experimental groups (n = 8), with measurements taken for body weight, serum glucose and triglycerides. Ovariectomized rats were used as a model of postmenopausal metabolic syndrome. At 8 weeks of age, female Wistar rats (purchased from Charles River Laboratories, Göttingen, Germany) were anesthetized with ketamine (70 mg/kg) and xylazine (10 mg/kg) administered intraperitoneally and then bilaterally ovariectomized using a midline incision (W-OVX). Sham-operated (W-sham) animals underwent the entire surgery, except for the removal of ovaries. Animals were saturated with oxygen throughout the procedure followed by subcutaneous analgesia (meloxicam 1 mg/kg). The health status of animals was monitored post-surgery. Food intake was measured weekly over a 4-month period to ensure the likely development of metabolic disorders associated with postmenopausal metabolic syndrome, as reported in previous studies [31].

At the end of the experiment, rats were sacrificed by decapitation after light anaesthetization (zoletil 5 mg/kg b.wt.) in a postprandial state. Aliquots of serum and tissue samples were collected and stored at −80 °C for further analysis.

### 4.2. Analytical Methods and Biochemical Analysis

Serum levels of triglycerides, glucose, total and HDL cholesterol, ALT, AST, GGT and non-esterified fatty acids (NEFA) were measured using commercially available kits (Erba Lachema, Czech Republic, Brno, and Roche Diagnostics, Mannheim, Germany). Serum insulin, HMW adiponectin, hs-CRP, MCP-1, IL-6, leptin and ghrelin concentrations were determined using the rat ELISA kit (Mercodia AB, Sweden; MyBioSource, USA; eBioscience, USA; BioVendor, Czech Republic). Serum 17β-estradiol and 17β-hydroxyprogesterone were analyzed using rat RIA kits (Immunotech, Prague Czech Republic).

For the oral glucose tolerance test (OGTT), blood glucose was determined after a glucose load (3 g of glucose/kg b.wt.) administered intragastrically after overnight fasting. Preceding the glucose load, blood was drawn from the tail at 0 min and then 30, 60 and 120 min thereafter.

To determine triglyceride and cholesterol content in tissues, samples were extracted using a chloroform/methanol mixture. The resulting pellet was dissolved in isopropyl alcohol, with triglyceride content determined by enzymatic assay (Erba-Lachema, Brno, Czech Republic).

### 4.3. Basal and Insulin-Stimulated Glucose Utilization in Adipose Tissue and Skeletal Muscles

For measurement of insulin-stimulated incorporation of glucose into lipids or glycogen, perimetrial adipose tissue or skeletal muscle was incubated for 2 h in 95% O_2_ + 5% CO_2_ in Krebs–Ringer bicarbonate buffer (pH 7.4) containing 0.1 μCi/mL of ^14^C-U glucose, 5 mmol/L of unlabeled glucose and 2.5 mg/mL of bovine serum albumin (Fraction V, Sigma, Czech Republic) with and without 250 μU/mL of insulin. Extraction of lipids or glycogen was followed by a determination of insulin-stimulated incorporation of glucose into lipids or glycogen [46]. In perimetrial adipose tissue, basal and adrenaline-stimulated lipolysis were measured ex vivo based on the release of NEFA into the incubating medium.

### 4.4. BAT Activity

Interscapular BAT metabolic activity was determined ex vivo based on the utilization of ^14^C-U-palmitic acid and ^14^C-U-glucose for oxidation (incorporation into CO_2_) and incorporation of ^14^C-U-palmitic acid and ^14^C-U-glucose into BAT lipids, as previously described. Lipolysis in interscapular BAT was measured ex vivo based on the release of NEFA into the incubating medium [47].

### 4.5. Oxidative and Dicarbonyl Stress Parameters

Levels of reduced (GSH) and oxidized (GSSG) forms of glutathione were determined using a high-performance liquid chromatography (HPLC) diagnostic kit with fluorescent detection (Chromsystems, Germany).

Methylglyoxal concentrations were measured using the same HPLC method with fluorescence detection after derivatization with 1,2-diaminobenzene [48]. Glo1 activity was analyzed according to a method described by Arai [49]. Activities of antioxidant enzymes, superoxide dismutase (SOD), glutathione peroxidase (GPx) and glutathione reductase (GR) were analyzed using Cayman Chemicals assay kits (Ann Arbor, MI, USA). Lipoperoxidation products were analyzed based on levels of thiobarbituric acid-reactive substances (TBARS).

### 4.6. Gene Expression Profile

Total RNA was isolated from tissues using RNA Blue (Top-Bio, Prague, Czech Republic). Reverse transcription and quantitative real-time PCR analysis was performed using the TaqMan RNA-to-C_T_ 1-Step Kit, TaqMan Gene Expression Assay (Applied Biosystems, Bedford, MA, USA) and ViiA^TM^ 7 Real-Time PCR System (Applied Biosystems, USA). Relative expression was determined after normalization against *Hprt* as an internal reference and calculated using the 2^−^^ΔΔCt^ method, with results run in triplicate.

### 4.7. Western Blotting and CYP4A Activity

Microsomes were prepared as previously described [50]. Samples containing 20 µg of lysed protein were electrophoresed on Mini-PROTEAN^®^ TGX^™^ gel (Bio-Rad Laboratories, Hercules, CA, USA), transferred to a PVDF membrane (Trans-Blot^®^ Turbo™ Midi PVDF Transfer Packs, Bio-Rad Laboratories, Hercules, CA, USA) using the Trans-Blot^®^ Turbo^™^ Transfer System (Bio-Rad Laboratories, Hercules, CA, USA), blocked in TBS-Tween and milk for 1 h and finally incubated with primary rabbit anti-CYP4A1/2/3 polyclonal antibody (Abcam, Cambridge, UK) and rabbit polyclonal anti-CYP2E1 antibody (Millipore, Temecula, CA, USA) overnight at 4 °C. For loading control, the mouse monoclonal anti-GAPDH antibody (Santa Cruz Biotechnology, Santa Cruz, CA, USA) was used. Membranes were then washed and incubated with the corresponding secondary antibody (Sigma-Aldrich, St. Louis, MO, USA). Proteins were detected using luminol reagent (WB, Luminol Reagent, Santa Cruz, CA, USA) and medical X-Ray films (Agfa, Belgium). For protein quantification, CanoScan Toolbox software (ver. 5.0) (Canon Europa, Amstelveen, The Netherlands) and ElfoMan software (ver. 2.6) (Semecky Inc., Prague, Czech Republic) were used.

Activity of CYP4A was analyzed using the P450-Glo assay kit with Luciferin-4A according to the manufacturer’s protocol (Promega, Madison, WI, USA). Luciferase activity was measured and calculated using the Infinite 200 Pro instrument (Tecan, Männedorf, Switzerland).

### 4.8. Statistical Analysis

All data are expressed as mean ± SEM. Before beginning the study, the χ^2^-square test was used to examine qualitative variables. Statistical data evaluation was performed using the unpaired Student’s *t*-test, with categorical variables analyzed using Fisher’s exac*t* test. Statistical significance was defined as *p* < 0.05. Statistical analysis was performed using BMDP Statistical Software.

## 5. Conclusions

Our results demonstrate that hepatic lipid dysmetabolism not only plays an important role in the early events of postmenopausal metabolic syndrome, but also precedes elevation of serum triglyceridemia, cholesterolemia and aggravated glucose tolerance. Impaired triglyceride regulation and secretion from the liver contributes to hepatic triglyceride accumulation in response to ovariectomy. In addition, hepatic lipid dysregulation reflects changes in the cytochrome P450 enzyme family, including CYP2E1, and contributes to hepatic steatosis development. Decreased protein and activity of CYP4A reflects changes in circulating hormones after ovariectomy and may lead to reduced fatty acid oxidation and hepatic steatosis. Reduced fatty acid utilization and lower lipolysis in brown adipose tissue may also influence the development of obesity and triglyceride accumulation during the postmenopausal period. These results should be used to promote further research with the aim of recommending preventative and therapeutic approaches in postmenopausal women.

## Figures and Tables

**Table 1:** Basal metabolic and morphological parameters in ovariectomized (W–OVX) rats compared to sham-operated control rats (W–sham).

	W–Sham	W–OVX	*p*˂
Initial body weight (g)	243 ± 18	253 ± 7	n.s.
Final body weight (g)	292 ± 24	346 ± 15	0.05
Visceral adipose tissue weight (g/100 g)	1.671 ± 0.325	1.908 ± 0.169	0.05
Brown adipose tissue weight (g/100 g)	0.075 ± 0.006	0.101 ± 0.009	0.05
Fasting glucose (mmol/L)	5.5 ± 0.2	5.9 ± 0.2	n.s.
Insulin (μmol/L)	0.190 ± 0.034	0.224 ± 0.027	n.s.
Glucagon (ng/mL)	0.182 ± 0.017	0.309 ± 0.083	n.s.
AUC_0–120_ (mmol/L)	708 ± 14	755 ± 24	n.s.
Serum triglycerides (mmol/L)	2.01 ± 0.36	1.80 ± 0.31	n.s.
Serum cholesterol (mmol/L)	1.82 ± 0.11	2.01 ± 0.08	n.s.
NEFA (mmol/L)	0.412 ± 0.045	0.568 ± 0.032	0.05
HDL-C (mmol/L)	1.28 ± 0.09	1.64 ± 0.06	0.05
17β-estradiol (pg/mL)	24.2 ± 2.4	9.5 ± 2.1	0.01
17β-hydroxyprogesterone (ng/mL)	1.28 ± 0.09	0.28 ± 0.11	0.001
hs-CRP (μg/mL)	523 ± 59	410 ± 41	n.s.
HMW adiponectin (μg/mL)	4.08 ± 0.87	3.81 ± 0.63	n.s.
Leptin (pg/mL)	3.41 ± 1.59	11.82 ± 1.47	0.001
Ghrelin (ng/mL)	0.044 ± 0.002	0.040 ± 0.003	n.s.
MCP-1 (ng/mL)	1.195 ± 0.268	1.908 ± 0.169	0.05
IL-6 (pg/mL)	40.97 ± 7.52	42.10 ± 2.97	n.s.

Data are mean ± SEM; n = 8 for each group.

**Table 2:** Hepatic parameters of lipid metabolism, transport, oxidative and dicarbonyl stress in ovariectomized (W–OVX) rats compared to sham-operated control rats (W–sham).

	W–Sham	W–OVX	*p*˂
Triglycerides in the liver (μmol/g)	5.44 ± 0.85	12.76 ± 1.10	0.001
Cholesterol in the liver (μmol/g)	8.98 ± 0.38	7.87 ± 0.64	n.s.
TBARS in the liver (μmol/mg prot)	1.54 ± 0.16	2.07 ± 0.15	0.05
Triglycerides in muscles (μmol/g)	3.79 ± 0.87	6.40 ± 0.55	0.05
Hepatic SOD (μmol/g)	0.130 ± 0.012	0.110 ± 0.004	n.s.
Hepatic GPx (mmol/L)	352 ± 17	237 ± 9	0.01
Hepatic GR (μmol/L)	116 ± 8	108 ± 8	n.s.
GSH in the liver (μmol/g protein)	63.32 ± 2.50	54.64 ± 6.64	n.s.
GSSG in the liver (μmol/g protein)	2.32 ± 1.43	3.71 ± 0.16	0.05
GSH/GSSG	28.36 ± 1.43	15.35 ± 1.29	0.01
ALT (μkat/L)	1.16 ± 0.04	1.22 ± 0.15	n.s.
AST (μkat/L)	2.74 ± 0.35	3.86 ± 0.47	0.05
GGT (μkat/L)	0.015 ± 0.002	0.025 ± 0.003	0.05

Data are mean ± SEM; n = 8 for each group.

## Data Availability

The data presented in this study are available in the article.

## Abbreviations

AGE	advanced glycation end-products
AUC_0–120_	the area under the curve during the oral glucose tolerance test
BAT	brown adipose tissue
CYP450	cytochrome P450
MCP-1	monocyte chemoattractant protein-1
NEFA	non-esterified fatty acid
NRF2	nuclear factor erythroid-2-related factor 2
GPx	glutathione peroxidase
GSH	reduced form of glutathione
GSSG	oxidized form of glutathione
PKC	protein kinase C
SOD	superoxide dismutase
SREBP	sterol regulatory element-binding protein
TBARS	thiobarbituric acid-reactive substance
TG	triglyceride
WAT	white adipose tissue
MG	methylglyoxal
Glo1	glyoxalase-1

## References

[1] Dorum A., Tonstad S., Liavaag A.H., Michelsen T.M., Hildrum B., Dahl A.A. (2008). Bilateral oophorectomy before 50 years of age is significantly associated with the metabolic syndrome and Framingham risk score: A controlled, population-based study (HUNT-2). Gynecol. Oncol..

[2] Lin W.Y., Yang W.S., Lee L.T., Chen C.Y., Liu C.S., Lin C.C., Huang K.C. (2006). Insulin resistance, obesity, and metabolic syndrome among non-diabetic pre- and post-menopausal women in North Taiwan. Int. J. Obes..

[3] Tawfik S.H., Mahmoud B.F., Saad M.I., Shehata M., Kamel M.A., Helmy M.H. (2015). Similar and additive effects of ovariectomy and diabetes on insulin resistance and lipid metabolism. Biochem. Res. Int..

[4] Shen M., Kumar S.P., Shi H. (2014). Estradiol regulates insulin signaling and inflammation in adipose tissue. Horm. Mol. Biol. Clin. Investig..

[5] Nigro M., Santos A.T., Barthem C.S., Louzada R.A., Fortunato R.S., Ketzer L.A., Carvalho D.P., de Meis L. (2014). A change in liver metabolism but not in brown adipose tissue thermogenesis is an early event in ovariectomy-induced obesity in rats. Endocrinology.

[6] Schilperoort M., Hoeke G., Kooijman S., Rensen P.C. (2016). Relevance of lipid metabolism for brown fat visualization and quantification. Curr. Opin. Lipidol..

[7] Ruiz J.R., Martinez-Tellez B., Sanchez-Delgado G., Osuna-Prieto F.J., Rensen P.C.N., Boon M.R. (2018). Role of Human Brown Fat in Obesity, Metabolism and Cardiovascular Disease: Strategies to Turn Up the Heat. Prog. Cardiovasc. Dis..

[8] Lee P., Greenfield J.R. (2015). Non-pharmacological and pharmacological strategies of brown adipose tissue recruitment in humans. Mol. Cell Endocrinol..

[9] DiStefano J.K. (2020). NAFLD and NASH in Postmenopausal Women: Implications for Diagnosis and Treatment. Endocrinology.

[10] Simpson A.E. (1997). The cytochrome P450 4 (CYP4) family. Gen. Pharm..

[11] Gao H., Cao Y., Xia H., Zhu X., Jin Y. (2020). CYP4A11 is involved in the development of nonalcoholic fatty liver disease via ROSinduced lipid peroxidation and inflammation. Int. J. Mol. Med..

[12] Wei Y., Wang D., Moran G., Estrada A., Pagliassotti M.J. (2013). Fructose-induced stress signaling in the liver involves methylglyoxal. Nutr. Metab..

[13] Bentley-Lewis R., Koruda K., Seely E.W. (2007). The metabolic syndrome in women. Nat. Clin. Pr. Endocrinol. Metab..

[14] Chalvon-Demersay T., Blachier F., Tome D., Blais A. (2017). Animal Models for the Study of the Relationships between Diet and Obesity: A Focus on Dietary Protein and Estrogen Deficiency. Front. Nutr..

[15] Brown L.M., Gent L., Davis K., Clegg D.J. (2010). Metabolic impact of sex hormones on obesity. Brain Res..

[16] Maliszewska K., Kretowski A. (2021). Brown Adipose Tissue and Its Role in Insulin and Glucose Homeostasis. Int. J. Mol. Sci..

[17] Heeren J., Scheja L. (2018). Brown adipose tissue and lipid metabolism. Curr. Opin. Lipidol..

[18] Kaikaew K., Grefhorst A., Steenbergen J., Swagemakers S.M.A., McLuskey A., Visser J.A. (2021). Sex difference in the mouse BAT transcriptome reveals a role of progesterone. J. Mol. Endocrinol..

[19] Quarta C., Mazza R., Pasquali R., Pagotto U. (2012). Role of sex hormones in modulation of brown adipose tissue activity. J. Mol. Endocrinol..

[20] Gonzalez-Garcia I., Tena-Sempere M., Lopez M. (2017). Estradiol Regulation of Brown Adipose Tissue Thermogenesis. Adv. Exp. Med. Biol..

[21] Orava J., Nuutila P., Lidell M.E., Oikonen V., Noponen T., Viljanen T., Scheinin M., Taittonen M., Niemi T., Enerback S. (2011). Different metabolic responses of human brown adipose tissue to activation by cold and insulin. Cell. Metab..

[22] Babaei P., Mehdizadeh R., Ansar M.M., Damirchi A. (2010). Effects of ovariectomy and estrogen replacement therapy on visceral adipose tissue and serum adiponectin levels in rats. Menopause Int..

[23] Laughlin G.A., Barrett-Connor E., May S. (2007). Sex-specific determinants of serum adiponectin in older adults: The role of endogenous sex hormones. Int. J. Obes..

[24] Jo D., Son Y., Yoon G., Song J., Kim O.Y. (2020). Role of Adiponectin and Brain Derived Neurotrophic Factor in Metabolic Regulation Involved in Adiposity and Body Fat Browning. J. Clin. Med..

[25] Yin C., Kang L., Lai C., Zhou J., Shi B., Zhang L., Chen H. (2017). Effects of 17beta-estradiol on leptin signaling in anterior pituitary of ovariectomized rats. Exp. Anim..

[26] Liu K., Liu P., Liu R., Wu X., Cai M. (2015). Relationship between serum leptin levels and bone mineral density: A systematic review and meta-analysis. Clin. Chim. Acta.

[27] Zidon T.M., Padilla J., Fritsche K.L., Welly R.J., McCabe L.T., Stricklin O.E., Frank A., Park Y., Clegg D.J., Lubahn D.B. (2020). Effects of ERbeta and ERalpha on OVX-induced changes in adiposity and insulin resistance. J. Endocrinol..

[28] Szendroedi J., Yoshimura T., Phielix E., Koliaki C., Marcucci M., Zhang D., Jelenik T., Muller J., Herder C., Nowotny P. (2014). Role of diacylglycerol activation of PKCtheta in lipid-induced muscle insulin resistance in humans. Proc. Natl. Acad. Sci. USA.

[29] Geisler C.E., Renquist B.J. (2017). Hepatic lipid accumulation: Cause and consequence of dysregulated glucoregulatory hormones. J. Endocrinol..

[30] Kitson A.P., Marks K.A., Aristizabal Henao J.J., Tupling A.R., Stark K.D. (2015). Prevention of hyperphagia prevents ovariectomy-induced triacylglycerol accumulation in liver, but not plasma. Nutr. Res..

[31] Medina-Contreras J., Villalobos-Molina R., Zarain-Herzberg A., Balderas-Villalobos J. (2020). Ovariectomized rodents as a menopausal metabolic syndrome model. A minireview. Mol. Cell. Biochem..

[32] Patel S.B., Graf G.A., Temel R.E. (2018). ABCG5 and ABCG8: More than a defense against xenosterols. J. Lipid Res..

[33] Poruba M., Anzenbacher P., Racova Z., Oliyarnyk O., Huttl M., Malinska H., Markova I., Gurska S., Kazdova L., Vecera R. (2019). The effect of combined diet containing n-3 polyunsaturated fatty acids and silymarin on metabolic syndrome in rats. Physiol. Res..

[34] DeBose-Boyd R.A., Ye J. (2018). SREBPs in Lipid Metabolism, Insulin Signaling, and Beyond. Trends Biochem. Sci..

[35] Ngo Sock E.T., Chapados N.A., Lavoie J.M. (2014). LDL receptor and Pcsk9 transcripts are decreased in liver of ovariectomized rats: Effects of exercise training. Horm. Metab. Res..

[36] Soffientini U., Caridis A.M., Dolan S., Graham A. (2014). Intracellular cholesterol transporters and modulation of hepatic lipid metabolism: Implications for diabetic dyslipidaemia and steatosis. Biochim. Biophys. Acta.

[37] Palmisano B.T., Zhu L., Stafford J.M. (2017). Role of Estrogens in the Regulation of Liver Lipid Metabolism. Adv. Exp. Med. Biol..

[38] Zhang Y., Klaassen C.D. (2013). Hormonal regulation of Cyp4a isoforms in mouse liver and kidney. Xenobiotica.

[39] Enriquez A., Leclercq I., Farrell G.C., Robertson G. (1999). Altered expression of hepatic CYP2E1 and CYP4A in obese, diabetic ob/ob mice, and fa/fa Zucker rats. Biochem. Biophys. Res. Commun..

[40] Leclercq I.A., Farrell G.C., Field J., Bell D.R., Gonzalez F.J., Robertson G.R. (2000). CYP2E1 and CYP4A as microsomal catalysts of lipid peroxides in murine nonalcoholic steatohepatitis. J. Clin. Investig..

[41] Zhang X., Li S., Zhou Y., Su W., Ruan X., Wang B., Zheng F., Warner M., Gustafsson J.A., Guan Y. (2017). Ablation of cytochrome P450 omega-hydroxylase 4A14 gene attenuates hepatic steatosis and fibrosis. Proc. Natl. Acad. Sci. USA.

[42] Jamwal R., Barlock B.J. (2020). Nonalcoholic Fatty Liver Disease (NAFLD) and Hepatic Cytochrome P450 (CYP) Enzymes. Pharmaceuticals.

[43] Huttl M., Markova I., Miklankova D., Makovicky P., Pelikanova T., Seda O., Sedova L., Malinska H. (2020). Adverse Effects of Methylglyoxal on Transcriptome and Metabolic Changes in Visceral Adipose Tissue in a Prediabetic Rat Model. Antioxidants.

[44] Neves C., Rodrigues T., Sereno J., Simoes C., Castelhano J., Goncalves J., Bento G., Goncalves S., Seica R., Domingues M.R. (2019). Dietary Glycotoxins Impair Hepatic Lipidemic Profile in Diet-Induced Obese Rats Causing Hepatic Oxidative Stress and Insulin Resistance. Oxid. Med. Cell. Longev..

[45] Matafome P., Sena C., Seica R. (2013). Methylglyoxal, obesity, and diabetes. Endocrine.

[46] Malinska H., Huttl M., Oliyarnyk O., Bratova M., Kazdova L. (2015). Conjugated linoleic acid reduces visceral and ectopic lipid accumulation and insulin resistance in chronic severe hypertriacylglycerolemia. Nutrition.

[47] Pravenec M., Landa V., Zidek V., Musilova A., Kazdova L., Qi N., Wang J., St Lezin E., Kurtz T.W. (2003). Transgenic expression of CD36 in the spontaneously hypertensive rat is associated with amelioration of metabolic disturbances but has no effect on hypertension. Physiol. Res..

[48] Thornalley P.J., Langborg A., Minhas H.S. (1999). Formation of glyoxal, methylglyoxal and 3-deoxyglucosone in the glycation of proteins by glucose. Biochem. J..

[49] Arai M., Nihonmatsu-Kikuchi N., Itokawa M., Rabbani N., Thornalley P.J. (2014). Measurement of glyoxalase activities. Biochem. Soc. Trans..

[50] Poruba M., Matuskova Z., Huttl M., Malinska H., Oliyarnyk O., Markova I., Gurska S., Kazdova L., Vecera R. (2019). Fenofibrate Decreases Hepatic P-Glycoprotein in a Rat Model of Hereditary Hypertriglyceridemia. Front. Pharm..

